# Collectin-11 promotes fibroblast proliferation and modulates their activation status and extracellular matrix synthesis

**DOI:** 10.3389/fimmu.2025.1592921

**Published:** 2025-08-14

**Authors:** Wan-Bing Chen, Bo Cao, Gang Li, Gang Wang, Kun-Yi Wu, Ting Zhang, Ning Ma, Wuding Zhou, Ke Li

**Affiliations:** ^1^ Department of Critical Care Medicine, The Second Affiliated Hospital, Xi'an Jiaotong University, Xi'an, China; ^2^ Core Research Laboratory, The Second Affiliated Hospital, Xi'an Jiaotong University, Xi'an, China; ^3^ Department of Urology, The Second Affiliated Hospital, Xi'an Jiaotong University, Xi’an, China; ^4^ Peter Gorer Department of Immunobiology, School of Immunology & Microbial Sciences, Faculty of Life Sciences & Medicine, King's College London, London,, United Kingdom

**Keywords:** collectin-11, renal fibroblast, cell proliferation, fibroblast function, EGFR and TGF-βRII

## Abstract

**Introduction:**

Collectin-11 (CL-11), a recently described soluble C-type lectin, has been shown to stimulate cell proliferation in fibroblasts and melanoma cells. However, its broader influence on fibroblast functions and the specific receptors mediating CL-11's effects remain to be elucidated.

**Methods:**

The EDU proliferation assay and WB detection of PCNA protein levels were used to evaluate the fibroblast proliferative effect after CL-11 stimulated. The qRT-PCR and WB detection of fibronectin and collagen I were used to evaluate the ECM production. The qRT-PCR detection of Il-6, Il-11, Tnfa, Il1b, Cxcl1, Egf, Tgfb1, Pdgfb were used to evaluate the cytokine production. The WB detection of ERK, AKT, STAT3, mTOR, SMAD2 and ACTIN were used to evaluate the activation of signaling pathway. The WB detection of ERK, AKT, STAT3, mTOR, SMAD2 and ACTIN were used to evaluate the activation of signaling pathway. The immunofluorescence and molecular docking experiments were used to detect the binding of CL-11 to EGFR/TGFRII.

**Results:**

In this study, we demonstrate that CL-11 not only promotes fibroblast proliferation but also modulates their activation status and extracellular matrix (ECM) synthesis. Specifically, treatment with recombinant CL-11 (rCL-11) significantly upregulated the production of ECM proteins (fibronectin, collagen I), growth factors (EGF, TGF-β1), and proinflammatory cytokines/chemokines (IL-6, TNF-α, IL-11, IL-1β, CXCL1) in renal fibroblasts. Additionally, rCL-11 activated multiple intracellular signaling pathways, including ERK, AKT/mTOR, STAT3, and SMAD2. Further, EGFR and TGF-βRII were found to be abundantly expressed in renal fibroblasts, and molecular docking analysis along with immunofluorescence/confocal microscopy confirmed CL-11's interaction with these receptors.

**Discussion:**

Our findings provide strong evidence that CL-11 plays a critical role in renal fibroblast proliferation and activation via engagement with EGFR and TGF-βRII, shedding light on the mechanisms by which CL-11 stimulates cellular activation and proliferation.

## Introduction

Collectins are a family of soluble C-type lectins, which represent an important group of pattern recognition molecules. Their primary structure is comprised of a carbohydrate-recognition domain, a neck region, triple-helical collagenous region, and N-terminal segment. Further oligomerization of this primary structure can give rise to more complex and multimeric structures. They are involved in a variety of immune functions, including recognition and binding to microorganisms, opsonization, and activation of the complement system (1, 2). Mannose-binding lectin and lung surfactant proteins are well known members among the group (3).

Collectin-11 (CL-11) is a recently described member of the collectin family. Due to its high expression in the kidney, CL-11 is also known as CL-K1. CL-11 is assembled into a homotrimeric subunit consisting of three polypeptide chains with a molecular mass of 100 KDa. CL-11 displays structural similarities with other collectins but has some distinct features such as having a wide tissue distribution, a relatively lower serum concentrations (~300 ng/mL) and binding a wide range of ligands (4–6). This suggests that CL-11 could potentially be involved in a wide range and different types of cellular processes through local action, and CL-11 produced by different tissues/cells can participates in those cellular processes. CL-11 is highly conserved among species; human and mice are 92% homologous at the amino acid level (4).

Previous studies have shown that CL-11 plays important roles in embryonic development and host defense (7, 8). CL-11 has also been shown to promote tumor growth, renal injury and renal fibrosis following renal ischemia reperfusion (9–11). Taken together, these findings suggest that CL-11 is a multifunctional molecule, not only playing roles in homeostasis and host defense but also participating in the pathogenesis of inflammatory and immunological diseases.

Fibroblasts are an important cell type involved in both tissue repair and wound healing, as well as in tissue fibrosis (12, 13). Fibroblasts are the most common type of cell found in connective tissue. They are responsible for producing and maintaining the extracellular matrix (ECM), which is the scaffold that provides structural support for tissues and organs (14, 15).In addition, by releasing signaling molecules, fibroblasts can influence their own behavior and the behavior of other cells in their environment through autocrine and paracrine regulation. Fibroblasts can become activated in response to stimuli (e.g. stress, hyperglycemia or hypoxia in renal disease). Activated fibroblasts produce a variety of inflammatory cytokines and chemokines, which recruit and activate other immune cells to the site of inflammation (16–18). Activated fibroblasts can also differentiate into myofibroblasts, which are specialized fibroblasts that produce large amounts of ECM and contribute to tissue fibrosis (19). Given that fibroblasts can switch between different phenotypes, from pro-repair to pro-fibrotic, and influence their own behavior and the behavior of other cells in their environment, therefore better understanding of functional modulation of fibroblasts is imperative, as which holds immense promise for therapeutic intervention in tissue fibrosis.

Our previous work in a murine model of renal ischemia/reperfusion injury has shown that CL-11 promotes the development of renal tubulointerstitial fibrosis. *In vitro*, it has been shown that CL-11 stimulates renal fibroblast proliferation (11). However, the effect of CL-11 on other functions of renal fibroblasts and the molecular mechanisms involved in the action of CL-11 are presently unknown. Such information will improve our understanding of diverse effects of CL-11 in renal fibroblasts and how CL-11 could contribute to renal fibrosis through regulating renal fibroblast functions. In this study, we employed primary renal fibroblast cultured from murine renal cortex to investigate: i) the role of CL-11 in regulating fibroblast behavior and functions (i.e. cell proliferation, ECM, cytokine/chemokine and growth factor production), ii) the involved signaling pathways, and iii) the potential receptors for CL-11 binding and acting. We also evaluated the role of CL-11 in human fibroblasts.

## Methods and materials

### Reagents

The following antibodies were used in signaling pathway studies: anti-phospho-ERK1/2 (Thr202/Tyr204, 4377), anti-phospho-Akt (Ser473, 4060), anti-phospho-STAT3 (Tyr705, 9145), anti-phospho-mTOR (Ser2448, 2971) anti-phosphor-SMAD2 (Ser465/467)/SMAD3 (Ser423/425) (8828), anti-ERK1/2 (4695), anti-Akt (9272), anti-STAT3 (4904), anti-SMAD2/3 (8685), β-Actin (4970) all are from Cell Signaling Technology. The following antibodies were used in protein detection by western blotting: anti-PCNA antibody (sc-56, Santa Cruz Biotechnology), GAPDH polyclonal antibody (10494-1-AP, Proteintech), β-Actin (4970, CST), COL1A1 Antibody (84336, CST), Anti-TGF beta 1 antibody (ab215715, Abcam), Anti-Fibronectin antibody (ab2413, Abcam), Peroxidase Goat Anti-Rabbit IgG (H+L) (33101ES60, Yeasen Biotechnology), Peroxidase AffiniPure Donkey Anti-Mouse IgG (H+L) (34101ES60, Yeasen). The following antibodies were used in immunofluorescence microscopy: anti-HER1 (EGFR) (D38B1, CST, mouse), anti-TGF beta Receptor 2 (D-2, Santa Cruz, mouse), rabbit (DA1E) mAb IgG XP Isotype Control(3900, CST), mouse IgG2b, kappa monoclonal Isotype Control(ab170192, abcam), anti-Vimentin (EPR3776, Abcam, mouse); Alexa Fluor^®^ 488 AffiniPure Goat Anti-Rabbit IgG (H+L) (33106ES60, Yeasen), Alexa Flour 488 AffiniPure Donkey anti-Mouse IgG (H+L) (34106ES60, Yeasen), Alexa Fluor 594 AffiniPure Rabbit Anti-Goat IgG (H+L) (33712ES60, Yeasen). We also used the following reagents: DMEM/F12 cell culture medium, Fetal Bovine Serum, PBS, 0.25% Trypsin EDTA, methyl alcohol, 4% paraformaldehyde (PFA), TRIzol, NuPAGE LDS Sample Buffer (4×), BSA, DAPI, RNaseA, HRP substrate, BCA protein assay kit, Alexa Fluor 488 Click-iT EdU Cell Proliferation Kit (catalog C10337) and Alexa Fluor 555 Tyramide SuperBoost Kit (catalog B40933) (all from Thermo Fisher Scientific). TB Green Permix Ex Taq TM II, ROX Referance Dye, 5×PrimeScript TM RT Master Mix, 100 bp DNA Ladder, M-PER mammalian protein extraction reagent, Premix Taq (all from Takara Bio). Recombinant CL-11 (rCL-11), including the mouse version (used for mouse cells) and the human version (used for human cells), as well as biotinylated rCL-11 (Bio-rCL-11, used in CL-11 binding assays, which is produced through genetic encoding of a biotin tag followed by *in vivo* enzymatic biotinylation), were obtained from Novoprotein. Biotinylated BSA (Bio-BSA) was from Beijing Psaitong Biotechnology. BSA and L-fucose were sourced from Sigma-Aldrich, EGFR siRNA (sc-29302, Santa Cruz), TGFRII siRNA (Suzhou GenePharma), control siRNA (sc-37007, Santa Cruz), siRNA Transfection Medium (sc-36868, Santa Cruz), siRNA Transfection Reagent (sc-29528, Santa Cruz).

### Human kidney and skin specimens and ethics

Normal human kidney tissue specimens were obtained from the unaffected portion of nephrectomized kidneys of patients who underwent surgery for renal tumors. All patients provided informed consent, and the study was conducted in compliance with the ethical guidelines outlined in the Declaration of Helsinki. The research protocol received approval from the Hospital Research Ethics Committee, ensuring that the study adhered to all relevant ethical standards for the use of human tissue samples in research.

### Cell cultures

Renal fibroblast cultures were prepared from kidneys of 8-12-week-old C57BL/6 mice as described previously (11). In brief, minced kidney cortex was digested with 0.1% collagenase II in DMEM/F-12 and passed through a series of sieves (mesh diameters of 250, 160, 75 and 40 µm). The filtered cells were collected and cultured in a DMEM-12 medium containing 10% FBS and 100 U/ml penicillin and 100 μg/ml streptomycin for 6–7 days. They then experienced at least three passages to eliminate contaminated epithelial cells. The final pure fibroblasts exhibited an elongated, spindle-shaped morphology with vimentin and fibronectin positive staining in immunofluorescence (**Supplementary Figure 1**).

Human renal fibroblasts were isolated from renal cortical tissue as described previously (20). The tissue was cut into small fragments and digested with collagenase type II (750 U/ml) at 37°C for 15 minutes. After digestion, the cell suspension was filtered through a series of mesh sieves with diameters of 250, 160, and 75 µm. The filtered cells were collected and cultured in DMEM-12 medium supplemented with 10% FBS, 100 U/ml penicillin, and 100 µg/ml streptomycin for 6–7 days. To ensure the removal of epithelial cells, the cultured cells underwent at least three passages. The remaining pure fibroblast population displayed an elongated, spindle-shaped morphology characteristic of fibroblasts. Flow cytometry analysis confirmed that approximately 80% of the cells were positive for vimentin, a fibroblast marker (**Supplementary Figure 2**), validating the fibroblastic identity of the isolated cells.

### Proliferation assay

The cells were seeded on cover slips in a 24-well plate with 6×10 (4) cells per well for mouse renal fibroblasts and 2×10 (4) cells per well for human renal and cultured in DMEM/F12 medium with 10% FBS for 24 hours. Subsequently, the cells were cultured in DMEM/F12 medium with 5% FBS alone or containing rCL-11 for an additional 48 hours. Following this, the cells were incubated with medium containing 10 mM EdU (DMEM/F12 with 5% FBS) for 5 hours and then fixed in 4% paraformaldehyde (PFA) for 15 minutes. Incorporated EdU was detected using the Click-iT EdU Alexa Fluor 488 Imaging kit according to the manufacturer’s instructions. Images were captured using a Leica SP8 confocal microscope, with eight fields at 20× magnification for each sample. To quantify proliferation, DAPI-positive nuclei (representing the total number of cells) and EdU-positive nuclei (representing the proliferating cells) were counted using Image J software. The proliferation rate was calculated using the formula: proliferation rate = (number of EdU-positive nuclei/number of DAPI-positive nuclei) × 100%.

### Western blotting

Cell preparation: For signaling detection, mouse renal fibroblasts were seeded in a 6-well plate and cultured in DMEM/F12 medium supplemented with 10% FBS for 24 hours. Following this, the cells were serum-starved in DMEM/F12 containing 2% FBS for 3 hours to synchronize the cells. The cells were then treated with rCL-11 at concentrations of 0, 300, and 600 ng/ml for 15 minutes or rCL-11 (600 ng/ml) for varying durations (0, 5, 15, or 30 minutes). For detecting PCNA, fibronectin (FN), collagen I (COL-I), and TGF-β1, mouse renal fibroblasts were seeded in 6-well plates and cultured in DMEM/F12 medium with 10% FBS for 24 hours, followed by overnight incubation in DMEM/F12 containing 5% FBS. They were then treated with rCL-11 (600 ng/ml) or BSA (control) for 48 hours.

Cell Lysis: After treatment, the cells were lysed using a mammalian protein extraction reagent containing protease inhibitors. The lysis was performed on ice for 15 minutes. The cell lysates were centrifuged at 14,000 g for 15 minutes at 4°C to separate the soluble protein fraction from the debris. The supernatant was collected, and the protein concentrations were determined using a BCA protein assay kit following the manufacturer’s instructions.

Protein detection: Equal amounts of protein were loaded onto SDS-PAGE gels for electrophoresis to separate the proteins based on molecular weight. The separated proteins were transferred from the gel to a polyvinylidene fluoride (PVDF) membrane for subsequent immunoblotting. The PVDF membranes were blocked with 5% BSA for 1 hour to prevent non-specific binding of antibodies. The membranes were incubated overnight at 4°C with specific primary antibodies. The next day, the membranes were washed and incubated with HRP-conjugated secondary antibodies for 1 hour at room temperature. Protein bands were visualized using the Amersham ECL Select detection reagent (GE Healthcare Life Sciences). Band intensities were quantified using ImageJ software. The relative amounts of phosphorylated signaling proteins (p-AKT, p-ERK, and p-STAT3) were normalized to the total protein levels of their respective proteins. The levels of PCNA and mTOR were normalized to β-actin (a loading control).

### Cell cycle analysis (PI staining)

Mouse renal fibroblasts were seeded in a 6-well plate and cultured in DMEM/F12 medium with 10% FBS for 24 hours. Subsequently, the cells were incubated in medium with DMEM/F12 containing 5% FBS alone or containing recombined mouse CL-11 (600 ng/ml) for 48 hours. At the end of the rCL-11 treatment, the cells were trypsinized and washed twice with PBS. After fixation with 75% alcohol overnight, they were incubated with RNase enzyme at room temperature for 15 minutes. Propidium iodide (1 mg/ml) was then added and incubated for 5 minutes before testing.

### Flow cytometric analysis

Human renal fibroblasts were trypsinized and washed twice with PBS. The cells were then permeabilized using a permeabilization solution for 20 minutes. Following this, the cells were preincubated with an FcR blocking antibody (CD16/32) in 10% goat serum and incubated with rabbit anti-human antibodies or the appropriate isotype control antibodies at 4°C for 20 minutes. Subsequently, the cells were incubated with FITC-conjugated goat anti-rabbit secondary antibody at 4°C for 30 minutes. Flow cytometric analysis was performed using a Calibur Flow Cytometer (BD Biosciences) and analyzed with FlowJo software.

### Conventional RT-PCR and RT-qPCR

Total RNA was extracted from fibroblasts using TRIzol reagent according to the manufacturer’s instructions. Complementary DNA (cDNA) was synthesized in a 10 μl reverse-transcription reaction mix containing 500 ng of total RNA, 2 μl of PrimeScript™ RT Master Mix. The reaction was carried out under the recommended conditions for cDNA synthesis. The conventional PCR was conducted in a 25 μl reaction volume containing: cDNA corresponding to 0.25 μg of RNA, 12.5 μl of Green master mix, 2 μM of forward and reverse primers, 2 μM of GAPDH primer (as the internal control). The PCR conditions included denaturation at 94°C, annealing at 60°C, and extension at 72°C, with a total of 30 cycles. The amplified PCR products were electrophoresed on a 1.5% agarose gel and visualized under ultraviolet light. TB Green Permix Ex Taq™ II was used for RT-qPCR according to the manufacturer’s guidelines. The relative gene expression was calculated using the 2^–ΔΔCT^ method (21) and expressed as 2^–ΔΔCT^, where Ct is the cycle number at which fluorescence exceeds the threshold. ΔΔ(Ct)= [Δ (Ct)-testing samples - Δ (Ct)-control samples, Δ(Ct)= [Ct-target gene - Ct-reference gene (18s)]. The control sample consisted of unstimulated fibroblasts, while the test samples were fibroblasts stimulated with rCL-11. Primer sequences are provided in **Supplementary Table 1**.

### Cell siRNA transfection

Mouse renal fibroblasts were seeded in 6-well plates and cultured in DMEM/F12 medium supplemented with 10% FBS for 24 hours. EGFR siRNA (75 nM), TGFβRII siRNA (3 pooled siRNA, 25 nM each), or scrambled control siRNA (75 nM) were incubated with a transfection reagent according to the manufacturer’s instructions. The siRNA-transfection reagent complexes were then added to the cells and incubated for 6 hours in transfection medium. After the transfection, the medium was replaced with fresh DMEM/F12 containing 10% FBS, and the cells were cultured for an additional 24 hours. Transfected cells were subsequently used to verify the knockdown efficiency of EGFR and TGFβRII, perform proliferation assays, and assess ECM protein expression by Western blotting.

### Detection of CL-11 binding to fibroblasts

The cells were seeded on cover slips in a 24-well plate at a density of 6×10 (4) cells per well and cultured in DMEM/F12 medium with 10% FBS for 48 hours. The cells were then fixed with 4% PFA for 15 minutes at room temperature without permeabilization. Subsequently, the cells were incubated in Bio-BSA or with Bio-CL-11 (1200 ng/ml) at 4°C overnight. HRP-conjugated streptavidin was then applied and incubated for 1 hour at room temperature, followed by incubation with Alexa Fluor 555 Tyramide reagent for 6 minutes according to the manufacturer’s instructions. To block CL-11 binding, L-Fucose (2 mg/ml) was incubated with Bio-rCL-11 for 30 minutes before applying it in the binding experiments. The staining was examined using a confocal microscope (Leica SP8).

### Molecular docking

Homology modeling is commonly employed to establish reliable protein conformations. As the protein structures of CL-11, epidermal growth factor receptor (EGFR), and transforming growth factor-β receptor II (TGF-βRII) in mice have not been resolved, their 3D structures were retrieved from SWISS-MODEL (https://swissmodel.expasy.org/) (22). The selection was based on Global Model Quality Estimation (GMQE) score, QMEANDisCo global score (QMEAN), and sequence similarity. GMQE values range from 0 to 1, with higher values indicating better quality. QMEAN scores range from 0 to 4, with lower scores indicating higher reliability. A sequence identity of over 40% indicates high reliability of the predicted protein compared to the template protein (23) (**Supplementary Table 2**).

To ensure the accuracy of the docking results, proteins were prepared using PyMol. Water molecules were manually removed, and polar hydrogens were added. Prepared protein structures were submitted to the HDOCK web server (http://hdock.phys.hust.edu.cn/) to generate molecular docking prediction models for CL-11 (SMR ID: Q3SXB8)-EGFR (SMR ID: Q01279) and CL-11-TGF-βRII (SMR ID: Q62312). HDOCK results display the molecular docking conformations. Models were selected based on docking score and confidence score. A lower docking score indicates a higher likelihood of binding, while confidence scores range from 0 to 1, with values greater than 0.7 indicating higher binding likelihood. The top-ranked model from the top ten predicted results was selected, and its PDB format was downloaded (24). Visual representations of the docking structure were generated using PyMol, and the structures of hydrogen bonds and salt bridges were analyzed using LigPlot+ (25, 26).

### Detection of CL-11 binding to receptors

Immunofluorescence was performed to detect CL-11 binding to EGFR or TGFβRII in renal fibroblasts using the tyramine signal amplification system. This methodology enables the simultaneous detection of multiple proteins of interest in each tissue or cell section. The staining was performed according to the manufacturer’s instructions. In brief, the cells were seeded on cover slips in 24-well plates and cultured in DMEM/F12 medium with 10% FBS for 48 hours. The cells were washed three times with PBS and fixed with 4% PFA for 15 minutes. The cells were then incubated with Bio-rCL-11 (1200 ng/ml) at 4°C overnight. This was followed by incubation with rabbit anti-mouse EGFR (1:50) or anti-TGFβRII (1:50) at 4°C overnight, secondary antibody (peroxidase goat anti-rabbit IgG for EGFR and peroxidase goat anti-mouse IgG for TGF-βRII) at room temperature for 1 hour, HRP-conjugated streptavidin at room temperature for 1 hour, and Alexa Fluor 555 Tyramide reagent for 6 minutes. Images were taken with the Leica SP8 system under ×63 magnification. Z-stack scanning was performed on the cells, with an average cell thickness of 50 microns, and 25 layers were generally scanned. The specificity of EGFR and TGF-βRII detection was assessed by incubating the cells with isotype control antibody and secondary antibody (**Supplementary Figure 3**).

### Immunofluorescence microscopy

Immunofluorescence was performed to detect vimentin and FN expression in renal fibroblasts. The cells were seeded on cover slips in 24-well plates and cultured in DMEM/F12 medium with 10% FBS. After washing the cells three times with PBS, they were fixed with 4% PFA for 15 minutes. The cells were then permeabilized with 0.5% Triton X-100 for 20 minutes. Subsequently, the cells were incubated overnight at 4°C with primary antibodies (vimentin, 1:50; FN, 1:50) and then at room temperature for 1 hour with the appropriate secondary antibody. Images were captured using the Leica SP8 confocal microscope under ×20 magnification.

### Statistics

Data are shown as mean ± SEM, unless otherwise specified. An unpaired two-tailed t-test was used to compare the means of two groups. A paired t-test was used to compare the means of matched pairs. One-way ANOVA and two-way ANOVA were used to compare the means of more than two independent groups. All analyses were performed using GraphPad Prism 10 software, unless otherwise specified. A p-value of less than 0.05 was considered significant.

## Results

### CL-11 stimulates mouse renal fibroblast proliferation

**Figure 1:**
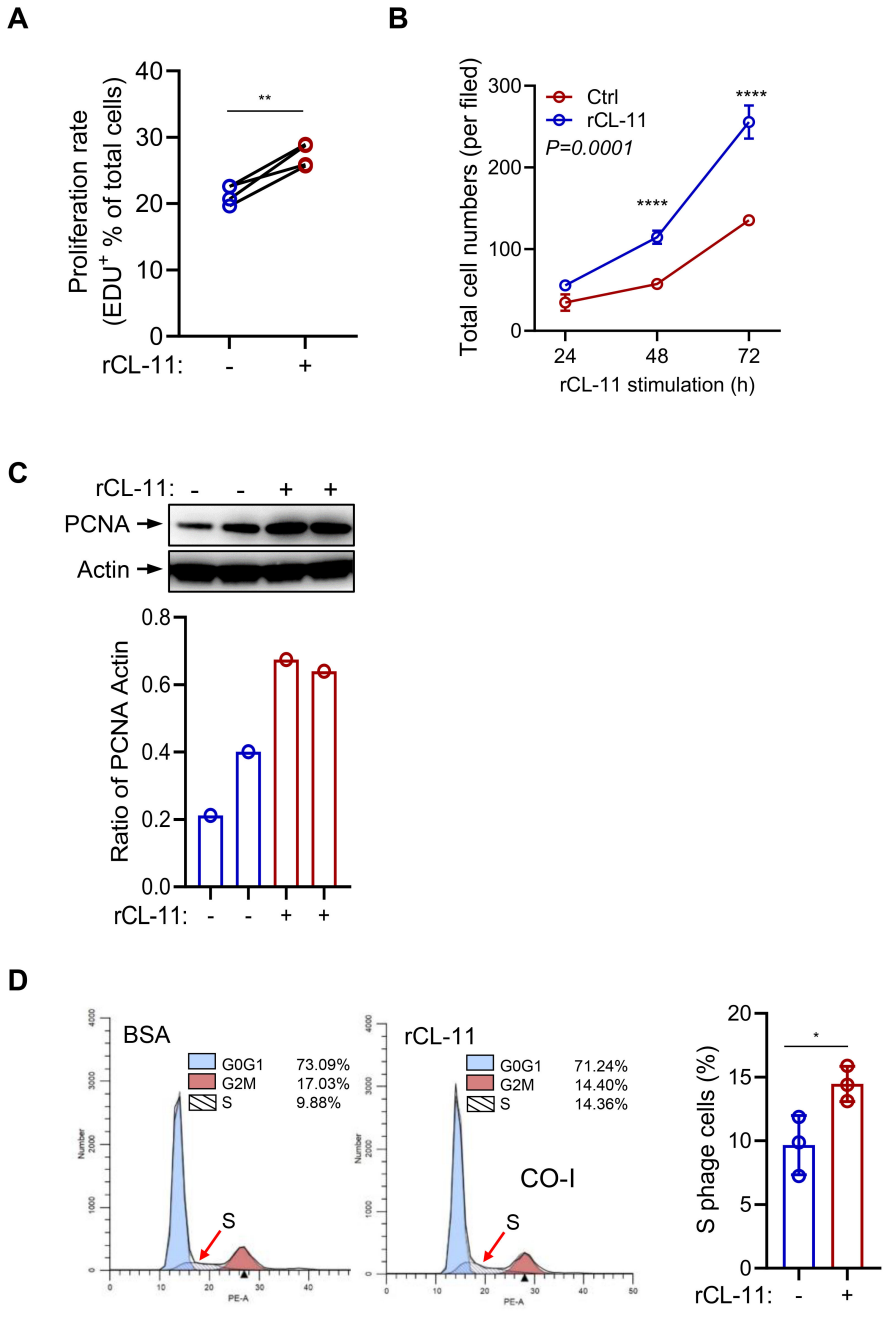
CL-11 stimulates mouse renal cell proliferation. **(A, B)** Cell proliferation in renal fibroblasts following rCL- 11 or control stimulation. **(A)** Cell proliferation rate (EDU+ cells) at 48 hours after rCL- 11 stimulation. Each data point is an average value of eight imaged fields. Data were analyzed by paired-test (n=4 independents experiments). **(B)** Total cell numbers (DAPI+ cells) at the indicated time points following rCL- 11 stimulation. Each data point is an average value of eight imaged fields. Data were analyzed by Two-way ANOVA with multiple comparisons (n=3 independent experiments). **(C)** Western blot analysis of PCNA in renal fibroblasts at 48 hours after rCL- 11 stimulation. Top panel: images of PCNA and actin blots. Bottom panel: Quantification of blots corresponding to the bolts in top panel. Representative results of 3 independent experiments are shown. **(D)** Flow cytometric analysis of PI staining in renal fibroblasts at 48 hours after rCL- 11 stimulation. Left and middle panels: representative flow graphs. Right panel: quantification of S phase cell population corresponding to the groups in left two graphs. Data were analyzed by unpaired t-test (n=3 iindependents experiments). *P<0.05; **P<0.005; ****P<0.0001.

To investigate the role of CL-11 in cell proliferation and functional modulation, we cultured primary mouse renal fibroblasts from the murine renal cortex. Immunochemical staining confirmed that 95% of the cells were positive for the fibroblast markers vimentin and fibronectin (FN) (**Supplementary Figure 1**). To evaluate the effect of CL-11 on renal fibroblast proliferation, an EdU proliferation assay was performed. Treatment with recombinant CL-11 (rCL-11, 600 ng/ml) significantly increased both the proliferation rate (EdU+ cells) and the total cell number (DAPI+ cells) compared to controls (**Figures 1A, B**). Further assessment of CL-11’s impact on cell proliferation was carried out via Western blotting, which revealed significantly elevated levels of proliferating cell nuclear antigen (PCNA) in rCL-11 treated cells compared to BSA-treated control cells (**Figure 1, C**). Additionally, flow cytometry analysis using propidium iodide (PI) staining demonstrated that rCL-11 treatment (600 ng/ml) significantly increased the proportion of cells in the S phase of the cell cycle, indicative of active DNA synthesis and cell proliferation (**Figure 1, D**). Collectively, these results demonstrate that CL-11 has a potent stimulatory effect on mouse renal fibroblast proliferation, suggesting a key role in fibroblast activation.

### CL-11 upregulates the expression of ECM and inflammatory mediators in mouse renal fibroblasts

**Figure 2:**
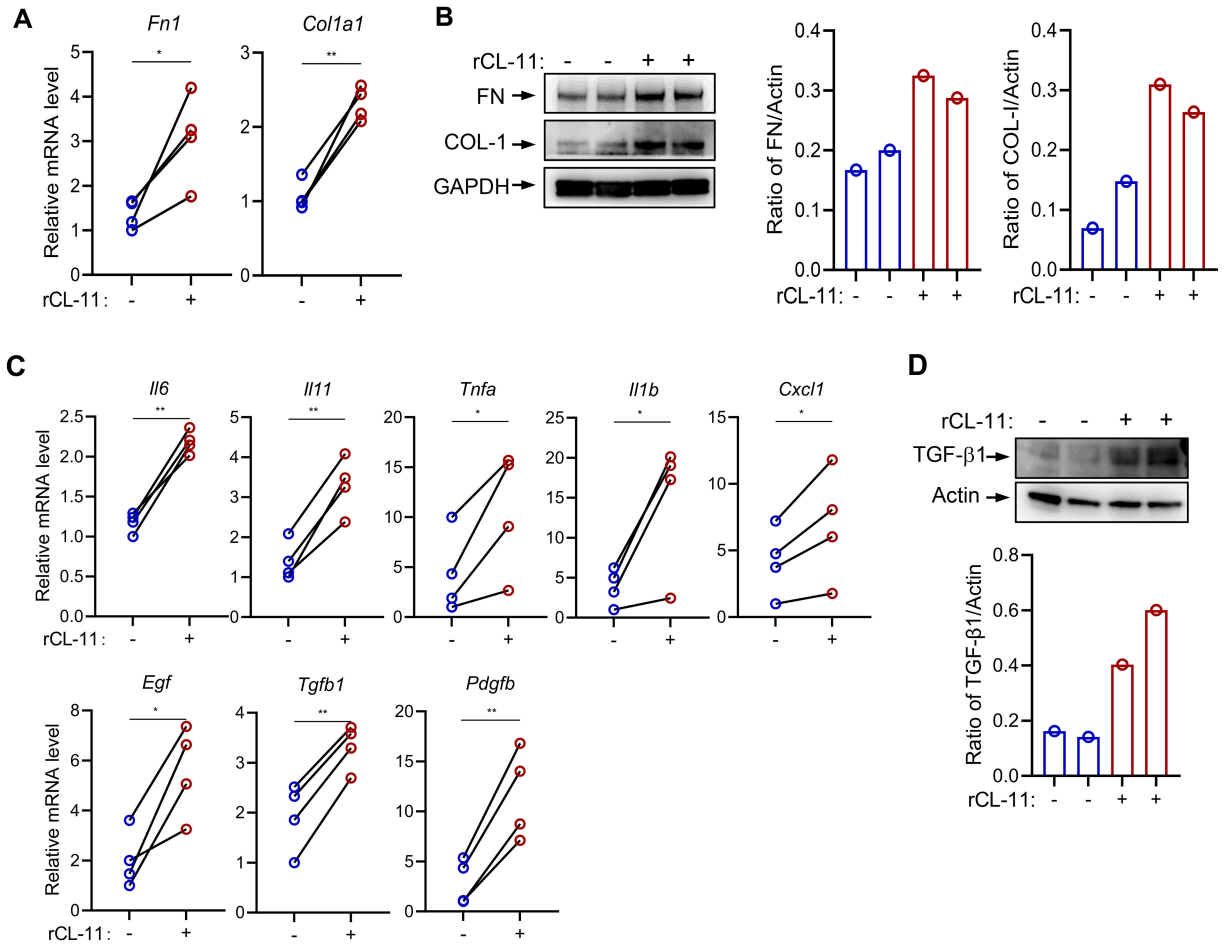
CL-11 modulates mouse renal fibroblast functions. **(A, B)** Fibro nectin (FN) and collagen l (COL-I) production in renal fibroblasts following rCL- 11 (600 ng/ml) stimulation for 48 hours. **(A)** RT-PCR analysisof mRNA levels. Data were analyzed by paired t-test (n=4 independent experiments). **(B)** Western blot analysis of protein levels in cell lysate. Left panel representative image of the blots. Middle and right panels: quantification of blots corresponding to the blots in left panel. Representative results of 2 independent experiments are shown. **(C)** RT-PCR analysis of cytokine and growth factor expression in fibroblasts following rCL- 11 (600 ng/ml) stimulation for 16 hours. Data were analyzed by pairedI-test (n=4 independent experiments). **(D)** Western blot analysis of FN and COL- 1 proteinlevels in cell lysate following rCL- 11 (600 ng/ml) stimulation for 48 hours. Top panel: representative image of the blots. Bottom panel: quantification of blots corresponding to the blots in left panel. Representative resultsof 3 indeperident experiments are shown. *P<0.05; **P<0.005.

Fibroblasts produce a variety of ECM proteins and factors involved in promoting a profibrotic myofibroblast phenotype, such as cytokines, chemokines, and growth factors. Therefore, we sought to determine whether CL-11 impacts the production of these molecules in primary mouse renal fibroblasts. Mouse renal fibroblasts were treated with rCL-11 (600 ng/ml). This treatment significantly upregulated both gene and protein expression of key ECM proteins, including fibronectin (FN) and collagen I (COL-I), as confirmed by RT-qPCR and Western blot (**Figures 2A, B**). Additionally, rCL-11 increased the gene expression of several pro-inflammatory cytokines and chemokines (IL-6, IL-11, TNF-α, IL-1β, CXCL1) and important growth factors (TGF-β, PDGF-β, EGF) involved in fibroblast activation and fibrosis (**Figure 2, C**). Western blot analysis further supported these findings, showing elevated TGF-β1 protein levels (**Figure 2, D**). Collectively, these results suggest that CL-11 promotes fibrogenic activity in renal fibroblasts by upregulating ECM components and inflammatory mediators, contributing to the profibrotic environment.

### CL-11 activates key cell proliferation and activation-related signaling pathways in mouse renal fibroblasts

**Figure 3:**
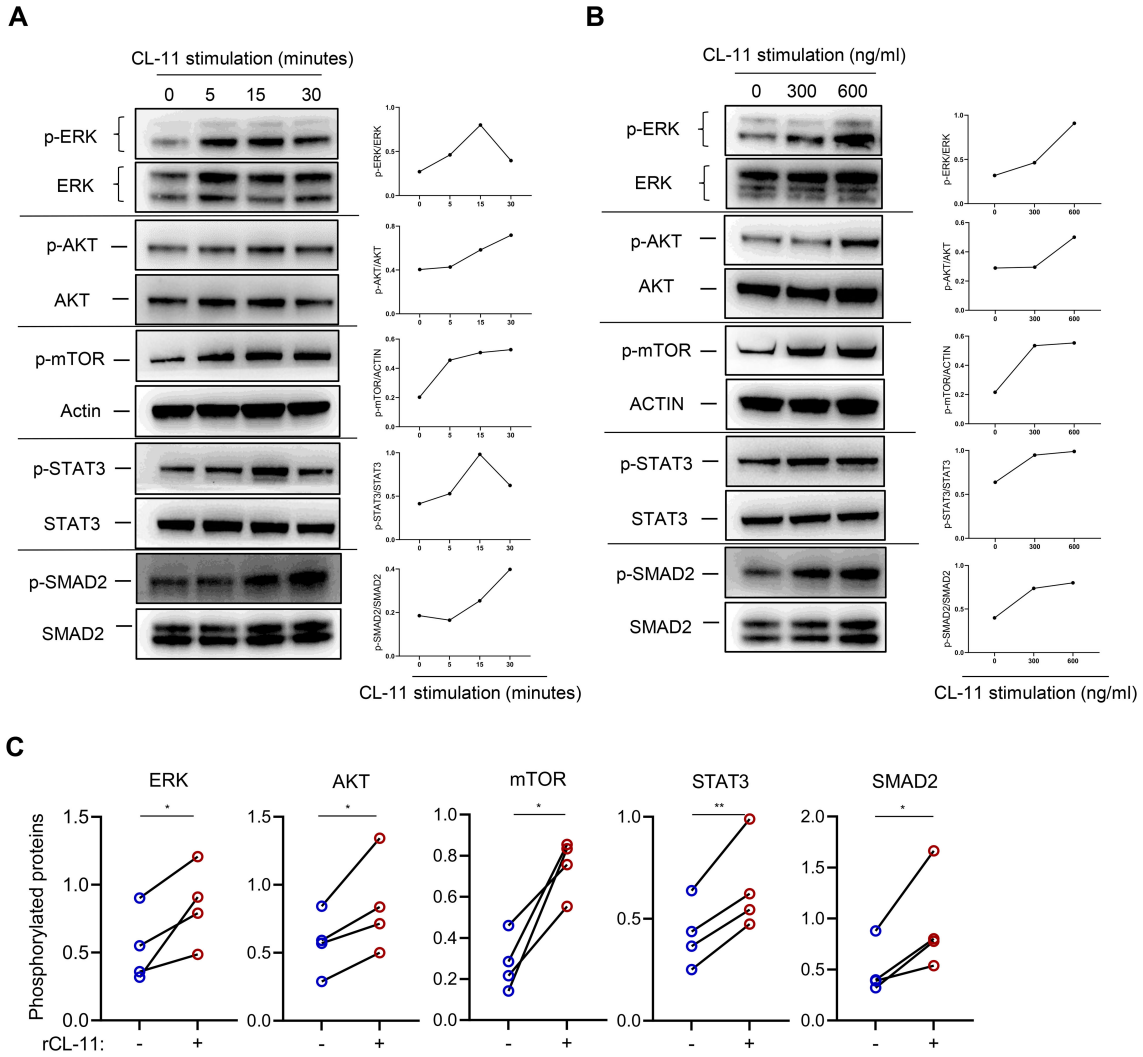
CL-11activates cell proliferation/activation-related signaling pathways in mouse renal fibroblasts. WB analysis of phosphorylated ERK, AKT, mTOR, STAT3, and SMAD2 in mouse renal fibroblasts following rCL-11 stimulation. **(A)** Analysis at different time points following rCL-11 (600 ng/ml) stimulation. Left panel: WB images for total and phosphorylated proteins.Right panel: Quantification of phosphorylated proteins relative to their respective total proteins or actin,corresponding to the blots in the left panel. Representative results of 2 independent experiments are shown. **(B)** WB analysis with different concentrations of rCL-11 stimulation for 15 minutes (or 30 minutes for SMAD2). Left panel: WB images for total and phosphorylated proteins. Right panel: Quantification of phosphorylated proteins relative to their respective total proteins or actin,corresponding to the blots in the left panel. Representative results of 4 independent experiments are shown. **(C)** Quantification of phosphorylated proteins relative to their respective total proteins or actin. Quantification resulting from 4 independent experiments with rCL-11 (600 ng/ml) stimulation for 15 minutes or 30 minutes for SMAD2. Data were analyzed by paired t-test (n=4 independent experiments). *P<0.05; **P<0.005.

Several signaling pathways, including Mitogen-activated protein kinase (MAPK), Phosphatidylinositol-3-kinase/AKT/mTOR (PI3K/AKT/MTOR), Janus kinase/signal transducer and activator of transcription (JAK/STAT3), and SMAD2/3, play essential roles in regulating cell proliferation, differentiation, and activation. To investigate the influence of CL-11 on these pathways, primary mouse renal fibroblasts were stimulated with rCL-11, 600 ng/ml for various time intervals. The results demonstrated that rCL-11 significantly increased the phosphorylation of ERK, AKT, mTOR, STAT3, and SMAD2 at 5, 15, and 30 minutes post-stimulation (**Figure 3, A**). A dose-dependent analysis further revealed that both 300 ng/mL and 600 ng/ml of rCL-11 enhanced the phosphorylation of these signaling molecules at the 15-minute time point (**Figures 3B, C**). These findings indicate that CL-11 is capable of activating multiple intracellular pathways associated with cell proliferation and fibroblast activation, emphasizing its potential role in promoting fibroblast function.

### CL-11 binds to mouse renal fibroblasts involving EGFR and TGF-βRII

After demonstrating that CL-11 promotes cell proliferation, modulates cell functions, and activates key intracellular signaling pathways in mouse renal fibroblasts, we sought to further investigate the underlying mechanism of CL-11 action. Specifically, we examined whether CL-11 directly binds to renal fibroblasts. To test this, renal fibroblasts were incubated with Bio-rCL-11 or Bio-BSA as a negative control. Immunofluorescence microscopy revealed the clear presence of CL-11 in cells treated with Bio-rCL-11, whereas no signal was detected in control cells.

**Figure 4:**
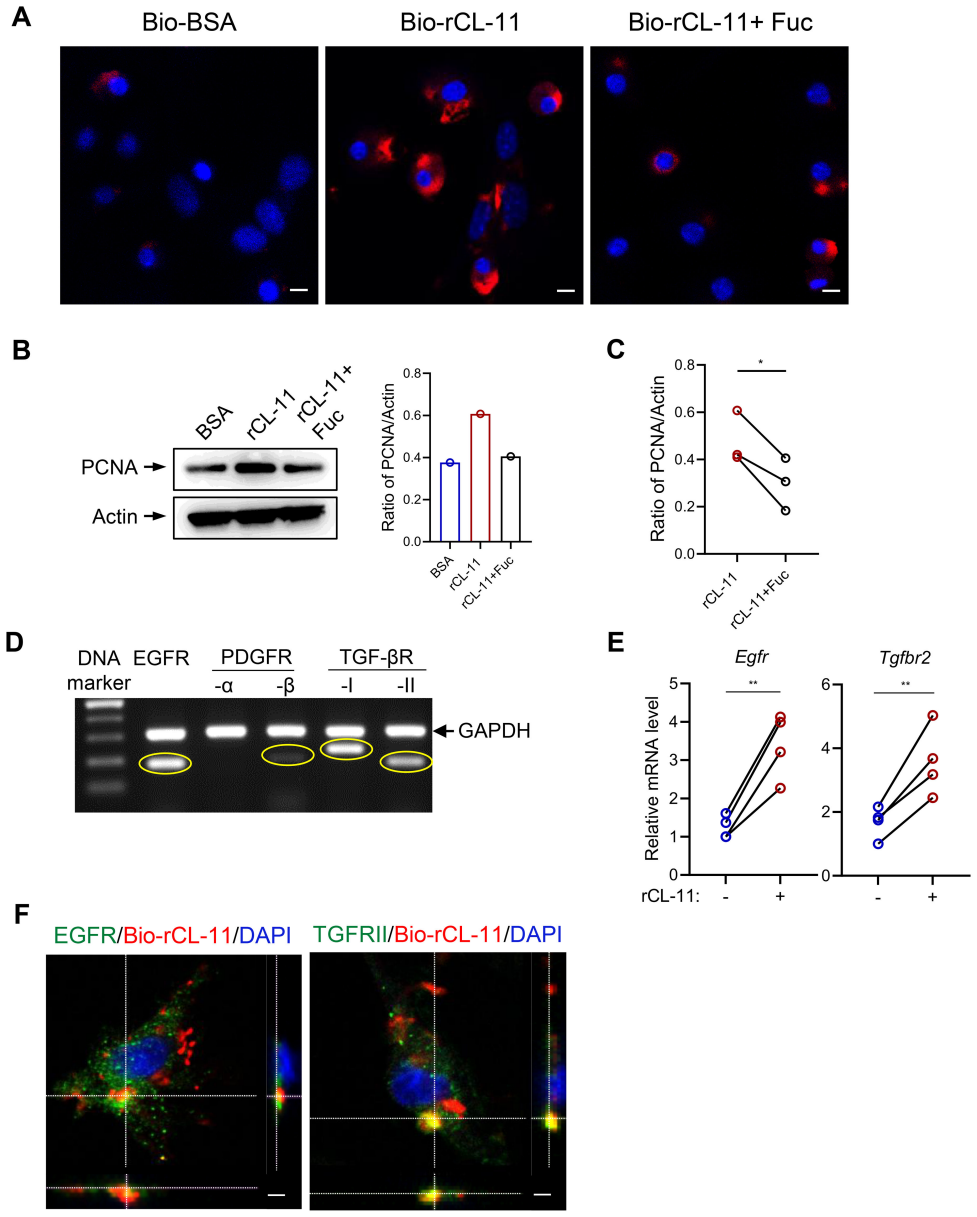
CL-11 binds to mouse renal fibroblasts involving EGFR and TGF-bRII. **(A)** lmmunoflourescene microscopic images of CL- 11 (red) in mouse renal fibroblasts that had been incubated with Bio- BSA (negative control), Bio-rCL- 11 or Bio-rCL- 11/L-Fucose. Nucei stained with DAPI (blue) are shown. Scale bar: 5 um. **(B)** Western blots analysis of PCNA in mouse renal fibroblasts that had been incubated with BSA, rCL- 11 or rCL- 11/L-Fucose. Left panel: Representative WB images of PCNA and actin blots. Right panel: Quantification of PCNA relative to actin, corresponding to the blots in the left panel. **(C)** Quantification of PCNA relative to actin. Data were analyzed by paired t-test (n=3 independent experiments). **(D)** The conventional RT-PCR and agarose gel electrophoresis to assess growth factor receptor expression in mouse renal fibrobtasts. **(E)** RT qPCR analysis of growth factor receptor expression in mouse renal fibroblasts following rCL- 11 (600 ng/ml) stimulation for 48 hours. Data were analyzed by paired t-test (n=4 independent experiments). **(F)** Representative microscopy Z- scan images showing Bio-rCL- 11 (red) colocalization with EGFR (green) and with TGF- bRII (green). DAPI (blue). Sea bar: 10 um. Fuc, L-Fucose; Bio-rCL- 11, Biotinylated recombined CL- 11; Bio- BSA, Biotinylated- BSA.*P<0.05; **P<0.005.

Pre-incubation of biotin-rCL-11 with L-fucose, a preferred ligand for CL-11, prevented its binding to renal fibroblasts (**Figure 4A**). Furthermore, blocking CL-11 binding with L-fucose significantly attenuated the increased proliferating cell nuclear antigen (PCNA) expression induced by rCL-11 (600 ng/ml) (**Figures 4B, C**). This finding highlights the importance of CL-11 binding in its role in cell proliferation.

Given the observation that CL-11 binds to the surface of renal fibroblasts and stimulates cell proliferation, we hypothesized that CL-11 mediates its effects through interactions with specific receptors. We focused on receptors known to be involved in fibrosis and cell proliferation. Using conventional PCR, we analyzed the expression of several receptors in mouse renal fibroblasts and found that EGFR and TGF-βRII were highly expressed, with PDGFR expressed to a lesser extent (**Figure 4D**). Additionally, rCL-11 stimulation upregulated the expression of EGFR and TGF-βRII, as confirmed by RT-qPCR (**Figure 4E**), indicating that these receptors may be involved in the CL-11-mediated signaling pathways that promote cell proliferation and activation. To confirm the interaction between CL-11 and these receptors, we performed fluorescence microscopy. Co-incubation of renal fibroblasts with Bio-rCL-11 and subsequent fluorescent labeling revealed that CL-11 binds to the surface of renal fibroblasts and colocalizes with EGFR and TGF-βRII (**Figure 4F**). This colocalization supports the hypothesis that CL-11 interacts directly with these receptors, mediating its effects on renal fibroblast proliferation and activation.

### CL-11 interacts with EGFR and TGF-βRII

**Figure 5:**
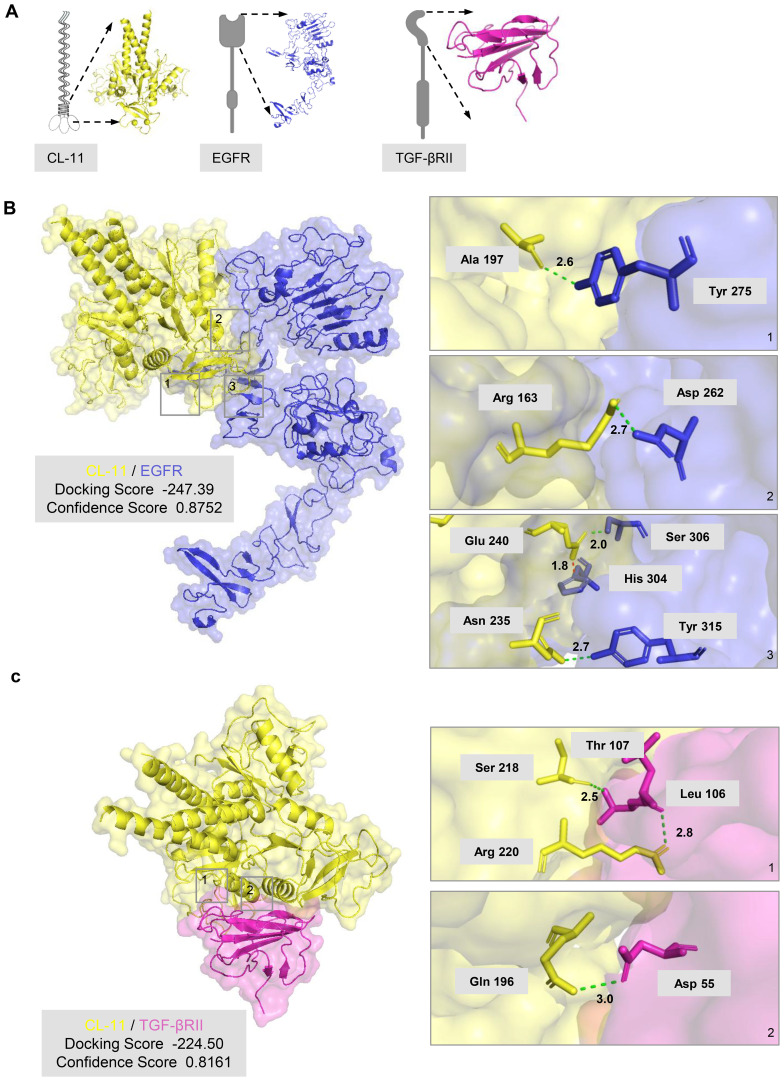
CL-11 interacts with EGFR and TGF-βRII **(A)** Three-<limensional model of trimeric mouse CL- 11 (yellow), wh r.eck arod carbohydrate- rerngnion domains (CRDs) highlighted, in compiex w h the extracellu lar regKrn of EGFR (blue) or the extracellular region of TGF- βRII (pink). **(B)** Predicted CL- 11- EGFR docking structure generated with HDOCK. The HDOCK binding score (lower values indicate stronger binding) and confidence score (>0.7 signifies high reliability) are shown. The three panels on the right are entarged views of the boxed regions on the ieft panel, labelling key interacting residues arod their inter-atomic: distances. Green dotted lines denote hydrogen borods; the red dotted line marks a salt bridge. **(C)** Predicted CL- 11- TGF- βRII docking structure generated with HDOCK. The HDOCK binding score (lower values indicate stronger binding) and confidence score (>0.7 signifies high reliability) are shown. The two panels on the right are entarged views of the boxed regions on the ieft panel, labelling key interacting residues and their inter-atomic: distances. Green dotted lines denote hydrogen bonds.

The cumulative data presented thus far suggest that CL-11 significantly influences renal fibroblast proliferation and functional modulation, primarily through its interactions with EGFR and TGF-βRII. To further investigate the potential for CL-11 to target EGFR and TGF-βRII, we performed molecular docking analysis using predictive protein structures generated by SWISS-MODEL (**Figure 5A**). The results indicated a high probability of protein binding for both the CL-11-EGFR and CL-11-TGF-βRII complexes. The CL-11-EGFR complex exhibited a high docking score of -247.39 and a confidence score of 0.8752, suggesting strong binding affinity. Specifically, the predicted binding sites and two-dimensional diagrams revealed four hydrogen bonds (CL-11-EGFR: Ala 197/Tyr 275, Glu 240/Ser 306, Asn 235/Tyr 315, and Arg 163/Asp 262) and one salt bridge (CL-11-EGFR: Glu 240/His 304) (**Figure 5B**). Similarly, the CL-11-TGF-βRII complex showed a high docking score of -224.50 and a confidence score of 0.8161. The binding affinities and two-dimensional diagrams revealed three hydrogen bonds (CL-11/TGF-βRII: Arg 220/Leu 106, Ser 218/Thr 107, and Gln 196/Asp 55) (**Figure 5C**).

**Figure 6:**
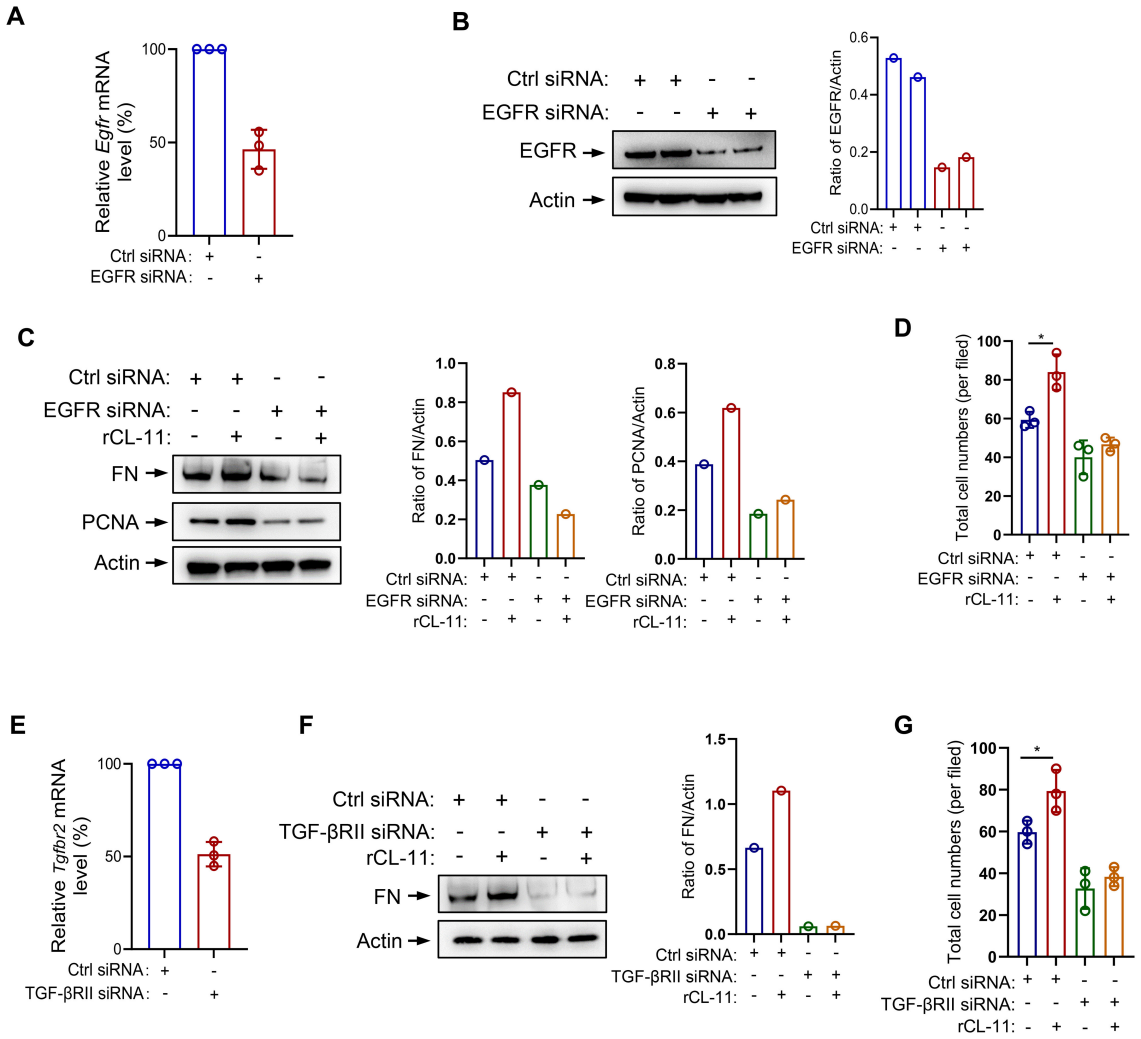
EGFR/TGF-βRII siRNA transfection in mouse renal fibroblast. **(A-D)** EGFR siRNA transfected mouse renal fibroblasts. **(A)** The effect of siRNA transfection on EGFR mRNA levels by RT-PCR (n=3 independent experiments). **(B)** The effect of siRNA transfection on EGFR protein levels by Western blot analysis. Left panel: Western blots. Right panel: quantification of the blots in the panel. **(C)** West blot analysis of FN and PCNA production following rCL- 11 (600 ng/ml) or control (BSA) stimulation for 48 hours after TGF-PRll siRNA transfection. Left panel: Western blots. Middle and right panels: quantification of the blots in the panel. Representative results of 2 independent experiments are shown. **(D)** Total cell numbers (DAPI+ cells) at 48hfollowing rCL- 11 stimulation after EGFR siRNA transfection. Each data point is an average value of six imaged fields (n=3 independent wells). (EG) TGF- PRll siRNA transfected mouse renal fibroblasts. **(E)** The effect of TGF- PRll siRNA transfection on TGF- PRll mRNA levels by RT- PCR (n=3 independents experiments) **(F)** Western blot analysis of FN production following rCL- 11 (600 ng/ml) or control (BSA) stimulation for 48 hours after TGF-βRII siRNA transfection. Left panel: Western blots. Right panel: quantification of the blots in panel. Representative results of 2 independent experiments are shown. **(G)** Total cell numbers (DAPI+ cells) at 48h following rCL- 11 stimulation after TGF-PRll siRNA transfection. Each data point is an average value of six imaged fields (n=3 independent wells). *P<0.05.

To assess whether CL-11 exerts its pro-proliferative and pro-fibrotic effects via these receptors, we performed loss-of-function experiments in mouse renal fibroblasts using siRNA-mediated knock-down of mouse EGFR or TGF-βRII. Transfection with EGFR-specific siRNA resulted in approximately ~50 % reduction in EGFR mRNA and ~70 % reduction in protein expression, as confirmed by RT-qPCR and Western blot analysis (**Figures 6A, B**). EGFR silencing effectively abolished the CL-11-induced increases in cell proliferation (including cell number and PCNA expression) and FN expression, reducing both to levels comparable to those observed in BSA-treated controls (**Figures 6C, D**). Transfection with 3 pooled TGF-βRII-specific siRNAs resulted in approximately ~50 % reduction in TGF-βRII mRNA expression, as confirmed by RT-qPCR (**Figure 6E**). TGF-βRII also silencing effectively abolished the CL-11-induced increases in cell proliferation and FN expression, reducing both to levels comparable to those observed in BSA-treated controls (**Figures 6F, G**). These data demonstrate that EGFR and TGF-βRII are required for CL-11-induced proliferation and extracellular-matrix activation in renal fibroblasts.

### CL-11 regulates the function of human fibroblasts

**Figure 7:**
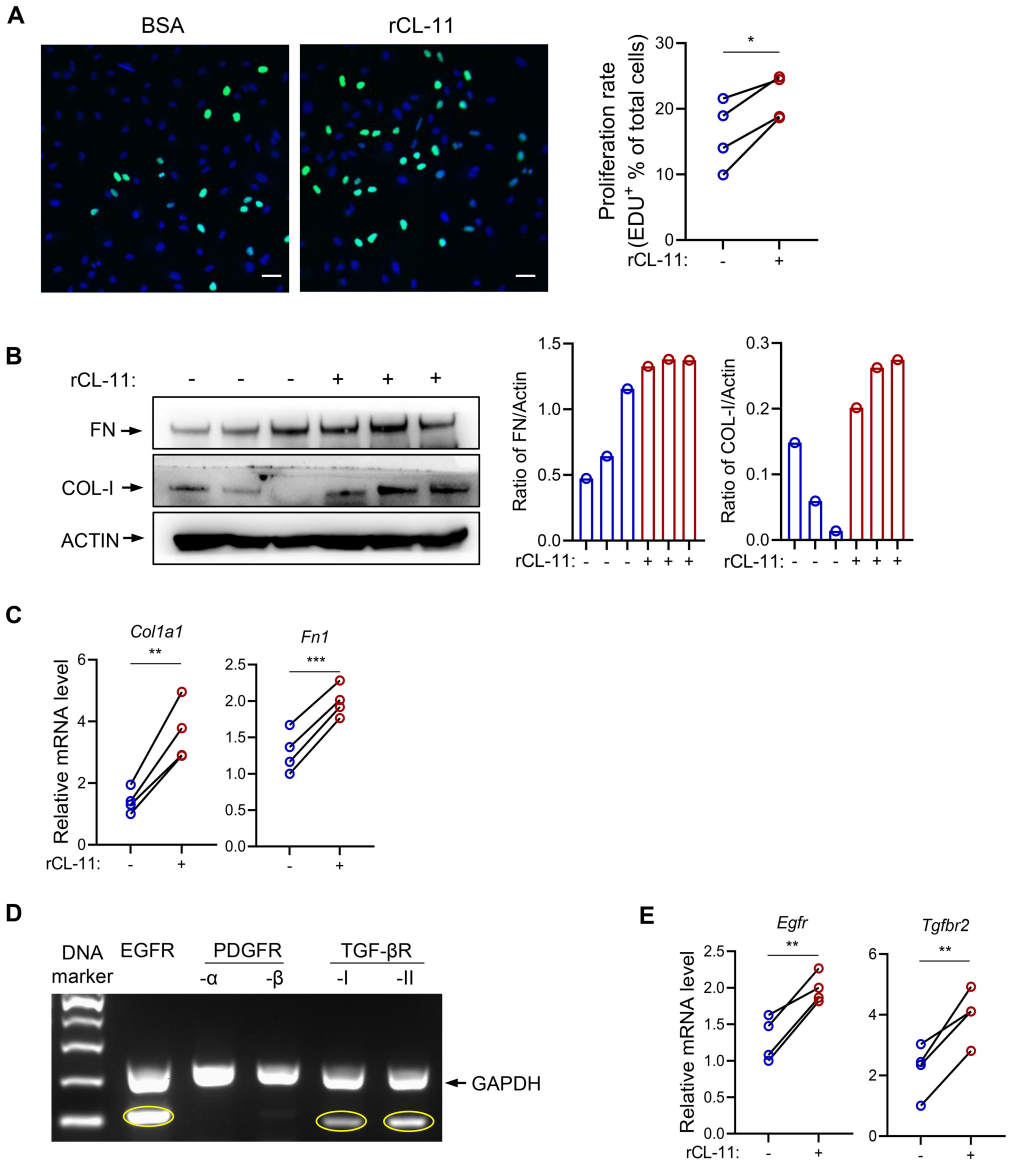
CL-11 affects human renal fibroblast function. **(A)** EDU labelling assay in human renal fibroblasts following BSA or rCL-11 stimulation for 48 hours.Left panel: lmmunofluorescence microscopy images showing EDU positive cells (20x ). Scale bar: 50 µm. Right panel: Quantification of cell proliferation rate corresponding to the two groups in the left panel. Each data point is an average value of eight imaged fields. **(B)** Western blot analysis of collagen I(COL-I) and fibronectin (FN) protein levels in cell lysate following BSA (control) or rCL-11 stimulation for 48 hours. Left panel: representative image of the blots. Right panel: Quantification of blots corresponding to the blots in the left panel. **(C)** RT-qPCR analysis of Collagen-I and Fibronectin expression in human renal fibroblasts following rCL-11 stimulation for 48 hours. **(D)** Convectional PCR detection of growth factor receptor expression in human renal fibroblasts. **(E)** RT-qPCR analysis of growth factor receptor expression in human renal fibroblasts following rCL-11 stimulation for 48 hours.Data in A,C and E were analyzed by paired t-test (n=4). *P<0.05; **P<0.005; ***P<0.001.

To further investigate the impact of CL-11 on fibroblast function, primary human renal fibroblasts were cultured. Using an EdU proliferation assay, we observed that rCL-11 treatment at 600 ng/ml significantly enhanced human renal fibroblast proliferation, as evidenced by the increased proportion of EdU+ proliferating cells (**Figure 7A**). WB and RT-qPCR analyses revealed that CL-11 upregulated the production of key ECM proteins (COL-I and FN) (**Figures 7B, C**), suggesting that CL-11 plays a role in ECM remodeling. Additionally, we explored the expression of two important receptors, EGFR and TGF-βRII, in human renal fibroblasts. The conventional PCR confirmed the high expression of both receptors (**Figure 7D**). Moreover, RT-qPCR analysis showed that stimulation with rCL-11 significantly increased the expression of EGFR and TGF-βRII in these cells (**Figure 7E**). These observations suggest that CL-11 may similarly regulate fibroblast function in human and mice.

## Discussion

Following our previous report of CL-11 promotes the development of renal tubulointerstitial fibrosis and renal fibroblast proliferation in a murine IR injury model, in the present study we sought to gain insights into the role of CL-11 in renal fibrosis and the mechanism of action of CL-11 in renal fibroblasts. Using primary renal fibroblast cultured from murine renal cortex, we demonstrate that CL-11 not only promotes cell proliferation but also upregulates the production of ECM and a range of cytokines/chemokines and several growth factors. Additionally, we also show that CL-11 induces the activation of signaling pathways involved in cell proliferation and activation (i.e. ERK, AKT/mTOR, STAT3, SMAD2). Furthermore, our study identifies that EGFR and TGFβRII are potential binding receptors for CL-11 in renal fibroblasts.

Renal fibroblasts are pivotal in the development of renal fibrosis, a common characteristic of progressive kidney diseases. These specialized cells are mainly responsible for synthesizing key ECM components like collagen, fibronectin, and various proteoglycans (27–29). This leads to the accumulation of scar tissue within the kidney, prompting tissue remodeling and a gradual decline in kidney function, ultimately culminating in renal failure. Beyond their direct involvement in ECM production and remodeling, renal fibroblasts exert an indirect influence by recruiting and activating other cell types, such as inflammatory and immune cells (30). Moreover, they produce a spectrum of profibrotic factors, including cytokines/chemokines and growth factors, further fueling the fibrotic process (15). Our findings clearly demonstrate that CL-11 stimulates cell proliferation and enhances ECM production, alongside upregulating the production of various cytokines/chemokines and growth factors in renal fibroblasts. This sheds light on the mechanisms through which CL-11 promotes renal fibrosis, contributing to a deeper understanding of the pathogenesis of this condition.

In the present study, we used three different methods, namely Edu staining, PCNA expression, and PI staining to determine the effect of CL-11 in renal fibroblast proliferation. EdU staining is commonly used to assess cell proliferation, which allows for the identification of cells that have synthesized DNA during a defined period. Detection of increased PCNA expressions in the cell samples following stimulation compared to controls indicates cell proliferation. PI staining is a common method used to assess cell viability and cell cycle distribution; S phase is the phase of the cell cycle in which DNA is replicated. Our findings show that treatment of renal fibroblasts with rCL-11 resulted in an increase of Edu+% cells, PCNA expression and the percentage of cells in the S phase, provided strong evidence supporting that CL-11 has stimulatory activity for the induction of renal fibroblast proliferation. This observation is aligned with previous findings in tumor cells (9). In addition to demonstrating the role of CL-11 in cell proliferation, we also show that CL-11 can upregulates the production of ECM (COL-I, FN), and a range of proinflammatory cytokines/chemokines (IL-6, TNF-α, IL-11, IL-1β, CXCL1) and three growth factors (EGF, TGF-β, PDGF-β) in the renal fibroblasts. Excessive ECM production can lead to the accumulation of scar tissue within the kidney, prompting tissue remodeling and a gradual decline in kidney function, ultimately culminating in renal failure (31). Proinflammatory cytokines and chemokines play a significant role in the development of renal fibrosis. They can act directly on renal cells to stimulate the production of ECM proteins and promote the apoptosis/death of renal tubular epithelial cells. Additionally, they can recruit other immune cells to the site of injury, which can further amplify the inflammatory response and ECM production (32). Growth factors (e.g. EGF, TGF-β, PDGF-β) can directly stimulate the production of ECM proteins by renal fibroblasts through engaging on growth factors on renal fibroblasts. Additionally, growth factor can promote the proliferation and migration of renal fibroblasts, which can further contribute to the development of renal fibrosis (33). Therefore, our findings that CL-11 stimulates cell proliferation and enhances ECM production, alongside upregulating the production of various cytokines/chemokines and growth factors in renal fibroblasts sheds light on the mechanisms through which CL-11 promotes renal fibrosis, contributing to a deeper understanding of the pathogenesis of this condition.

Another important finding of our study is the identification of serval signaling pathways that were induced by rCL-11, including ERK, AKT/mTOR, and STAT3. ERK signaling plays a crucial role in cell proliferation by regulating the expression of genes involved in cell cycle progression and DNA synthesis. In addition to its role in cell proliferation, ERK signaling is also involved in cell activation. For example, ERK signaling can activate the transcription factor NF-κB, which is involved in the regulation of genes involved in inflammation and immunity (34). The AKT/mTOR pathway is a major pathway that regulates cell proliferation and activation. It is activated by a wide variety of extracellular signals and can regulate the expression of a variety of genes involved in cell cycle progression, DNA synthesis, inflammation, immunity, cell differentiation, and apoptosis (35). STAT3 (signal transducer and activator of transcription 3) is a transcription factor that plays a key role in cell proliferation and activation by activating the transcription of genes involved in cell cycle progression (e.g. c-Myc, cyclin D1) and in inflammation (e.g. IL-6, TNF-α) (36, 37). Although SMAD2 is a core mediator of TGF-β signaling, its precise role in renal fibroblast activation remains incompletely defined and published animal studies have yielded seemingly conflicting results that vary with the disease model and targeted cell types. Global SMAD2 deletion in the unilateral ureteral obstruction model exacerbates renal fibrosis, an effect attributed chiefly to altered signaling in tubular epithelial cells (38). Conversely, fibroblast-specific protein-1 - driven SMAD2 knockout markedly attenuates fibrosis in streptozotocin-induced diabetic mice, implicating SMAD2 in profibrotic pathways within mesenchymal-derived cells (39). Consistent with the latter observations, we show here that CL-11 stimulation enhances SMAD2 phosphorylation in primary renal fibroblasts, supporting a model in which SMAD2 drives fibroblast proliferation and activation during renal fibrogenesis. Therefore, the identification of ERK, AKT/mTOR, STAT3 and SMAD2 induced by rCL-11 further support the observed effects of CL-11 on proliferation and functional regulation in renal fibroblasts. It also suggests that CL-11 stimulates renal fibroblasts through binding cell surface receptors.

In this study, we employed a range of methodologies to identify potential receptors involved in CL-11 binding and its subsequent functional effects on renal fibroblasts. Our cellular assays provided strong evidence that CL-11 binds to renal fibroblasts via interactions with EGFR and TGF-βRII. The observed upregulation of these receptors upon CL-11 stimulation, along with their colocalization with CL-11, highlights the crucial roles that EGFR and TGF-βRII play in mediating CL-11’s effects on renal fibroblasts. The findings that knockdown of EGFR or TGF-βRII expression effectively attenuated the effects of CL-11 on fibroblast proliferation and ECM production provide direct functional evidence supporting the roles of EGFR and TGF-βRII as mediators of CL-11 signaling in renal fibroblasts. Furthermore, molecular docking analysis confirmed specific interactions between CL-11 and these key receptors. The docking and confidence scores, coupled with detailed identification of hydrogen bonds, salt bridges, and interacting residues, provided in-depth insights into the strength and nature of these molecular interactions. Overall, these results, supported by signaling pathway assays, offer compelling evidence that CL-11 promotes renal fibroblast proliferation and regulates their functions primarily through its interaction with EGFR and TGF-βRII. This finding not only sheds light on the potential role of CL-11 in renal fibroblast biology but also opens new possibilities for understanding the molecular mechanisms underlying renal fibrosis and other fibroblast-related pathologies.

**Figure 8:**
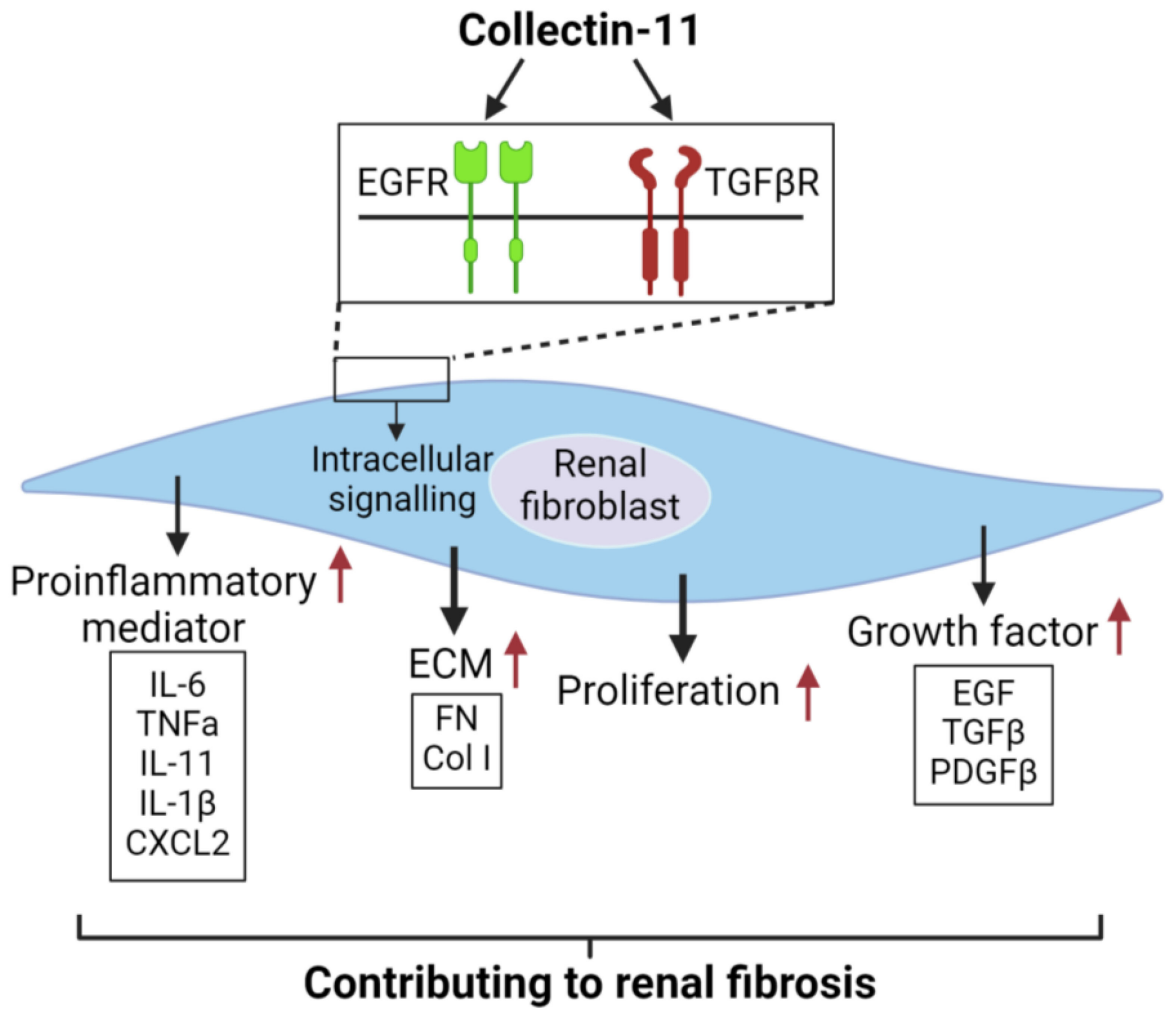
Proposed mechanism to describe the actions of CL-11 in renal fibroblasts .CL-11 binds to the cell surface receptors EGFR and TGFβRII, activates intracellutar proliferation-retated signaling pathways, and has a direct proliferative effect on fibroblasts by affecting their ability to secrete ECM and cytokines.

Based on our findings and literature interpretation, we propose a mechanism to describe the actions of CL-11 in renal fibroblasts (**Figure 8**). CL-11 interacts with EGFR and TGF-βRII to activate intracellular signaling pathways, including AKT/mTOR, ERK, STAT3, and SMAD2. This activation leads to cell proliferation and upregulation of ECM, growth factor, and proinflammatory mediator expression. These processes collectively contribute to renal fibrosis.

In the present study, we investigated the role of CL-11 in promoting fibroblast proliferation in mouse and human renal cells. Extending our work in mouse renal fibroblasts, we demonstrate that CL-11 similarly enhances cell proliferation, extracellular matrix production, and growth factor expression in primary renal fibroblasts. Moreover, both EGFR and TGF-β receptors I and II were detected in mouse fibroblasts, paralleling their expression in human cells. These findings suggest that the CL-11-mediated signaling pathways identified in mice are also active in human fibroblasts. Our results support the biological relevance of the CL-11–EGFR/TGF-β receptor axis in renal fibrosis and highlight its potential as a therapeutic target. Specifically, interrupting CL-11 signaling or blocking its interaction with EGFR and TGF-β receptors could offer a novel strategy to suppress fibroblast proliferation and fibrogenic activity - providing a promising avenue for the treatment of renal fibrosis and potentially other fibroproliferative disorders. Furthermore, assessing CL-11 levels in kidney biopsies or urine samples from patients with varying stages of chronic kidney disease may validate its clinical utility as a biomarker or therapeutic target.

Previous studies have implicated CL-11, EGFR, and TGF-β signaling in fibroblast proliferation and renal fibrosis in murine models (11, 40–42). Building on this foundation, our findings provide mechanistic insight into how CL-11 activates fibroblasts and drives ECM production via EGFR and TGF-β pathways. Collectively, these results support the hypothesis that CL-11–induced fibroblast activation through EGFR and TGF-β signaling constitutes a central pathogenic mechanism in renal fibrosis. However, the absence of *in vivo* validation remains a limitation. Future studies using animal models of renal fibrosis will be critical to confirm the pathogenic role of CL-11 in fibroblast activation and matrix remodeling *in vivo*.

In summary, our findings demonstrate that CL-11 is an important stimulus for renal fibroblasts, promoting cell proliferation and upregulating the production of ECM, proinflammatory cytokines/chemokines, and growth factors. Our findings also identify that EGFR and TGF-βRII are possible receptors for CL-11 binding and action on renal fibroblasts. Furthermore, our findings offer some insight into how CL-11 could promote renal tubulointerstitial fibrosis. This study highlights the potential of targeting CL-11 and its receptors as a therapeutic strategy in renal fibrosis and related pathologies.

## Data Availability

The raw data supporting the conclusions of this article will be made available by the authors, without undue reservation.
