# Supplementary figures and images for: Collectin-11 promotes fibroblast proliferation and modulates their activation status and extracellular matrix synthesis
Source: Front Immunol. 2025 Aug 14;16:1592921. doi: 10.3389/fimmu.2025.1592921 (PMC12391065; doi:10.3389/fimmu.2025.1592921)

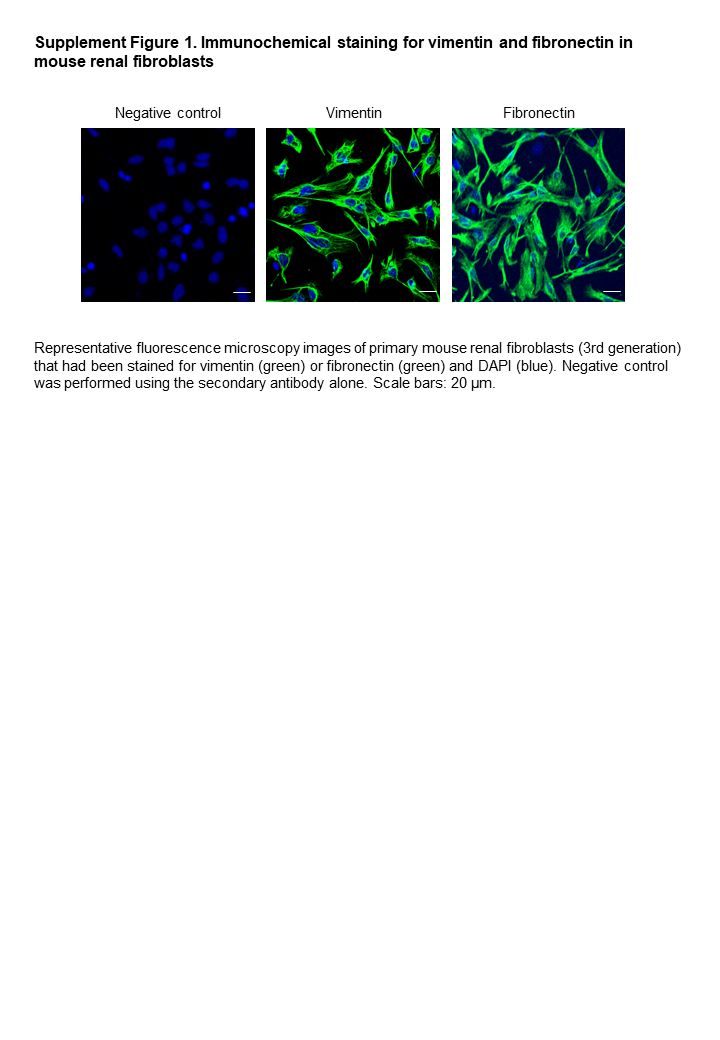

Supplement: Supplementary file 1 [file Image1.tif]

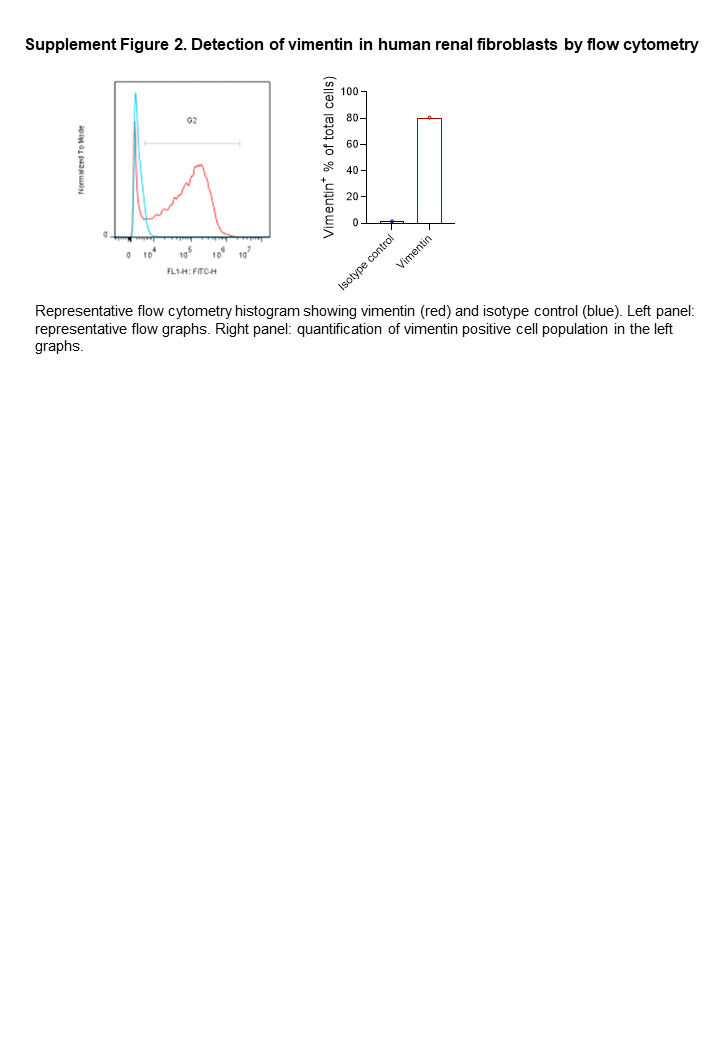

Supplement: Supplementary file 2 [file Image2.tif]

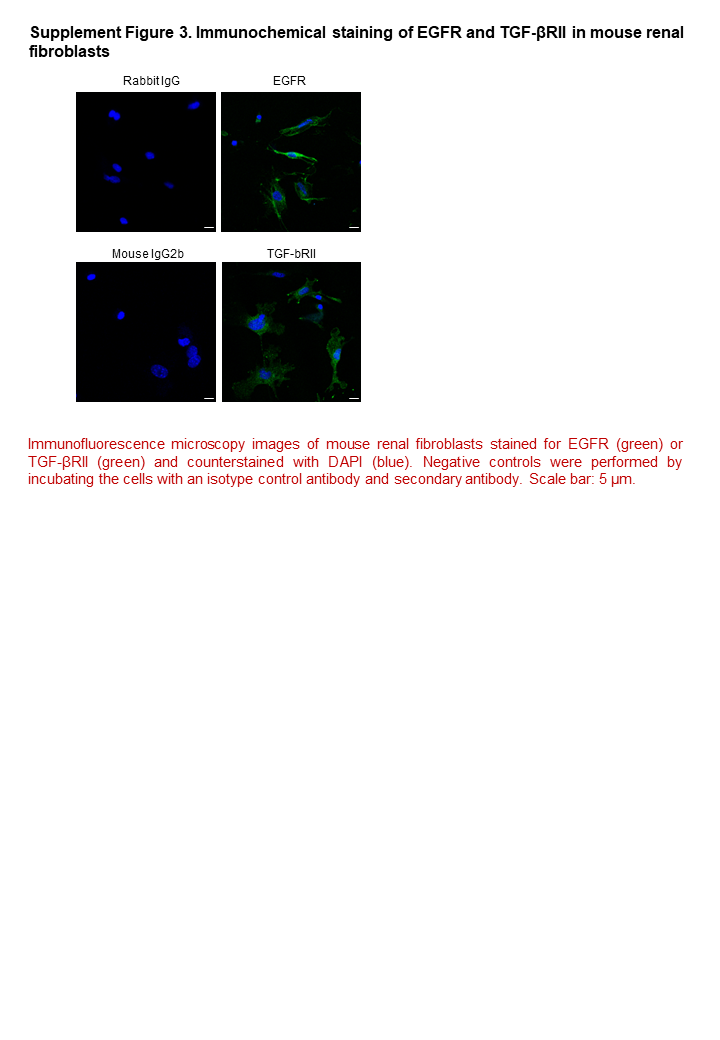

Supplement: Supplementary file 3 [file Image3.tif]

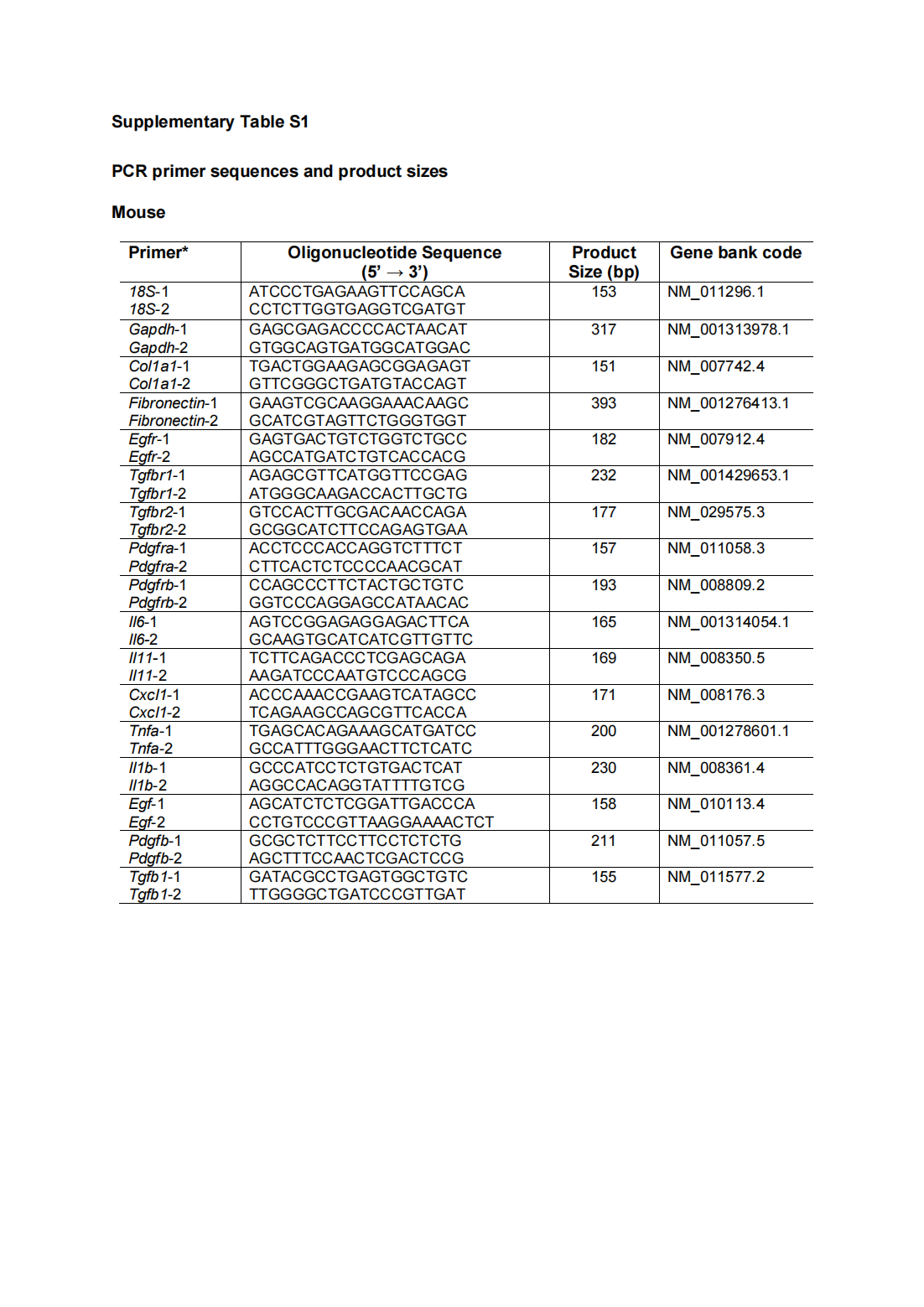

Supplement: Supplementary file 4 [file Image4.tif]

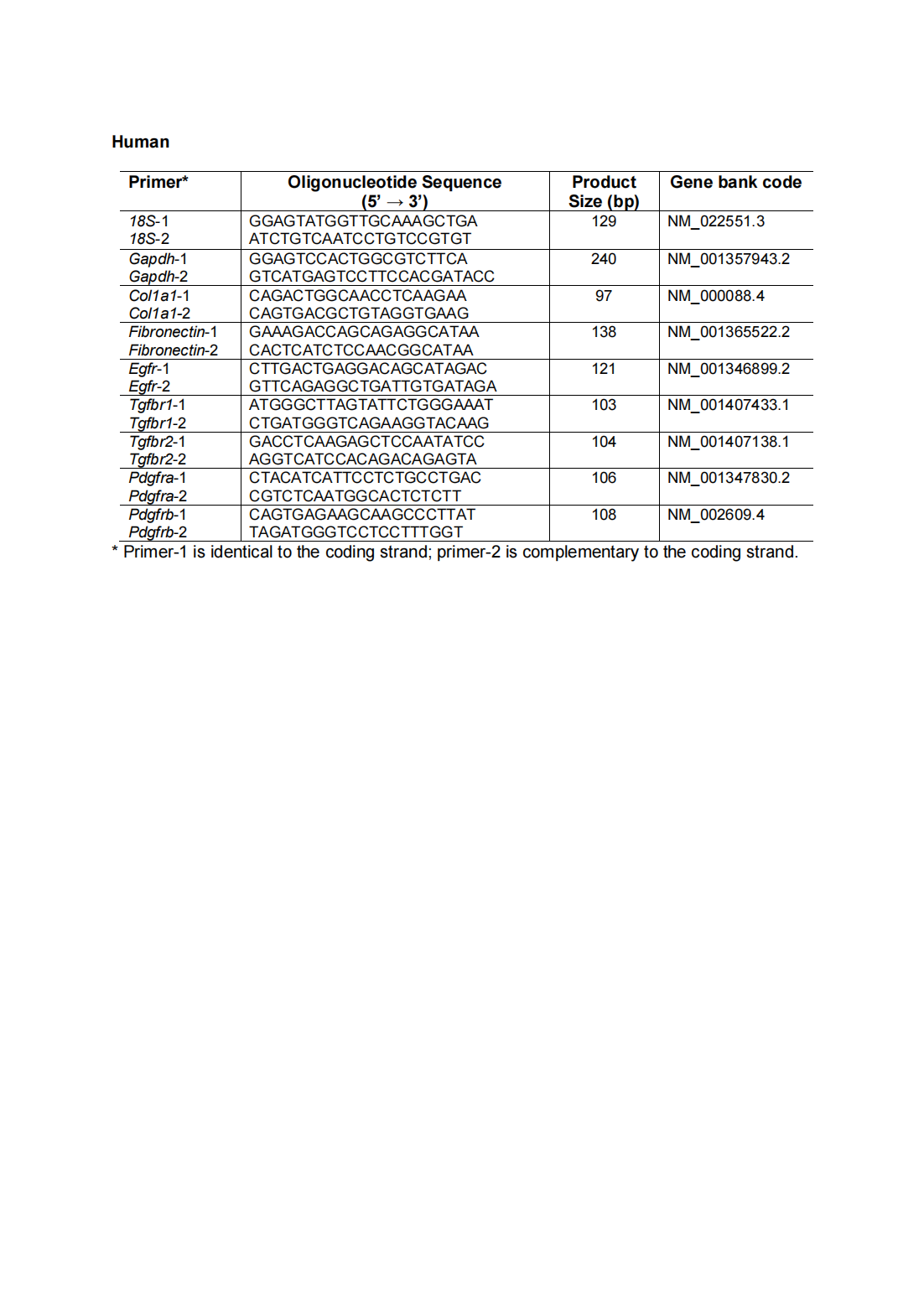

Supplement: Supplementary file 5 [file Image5.tif]

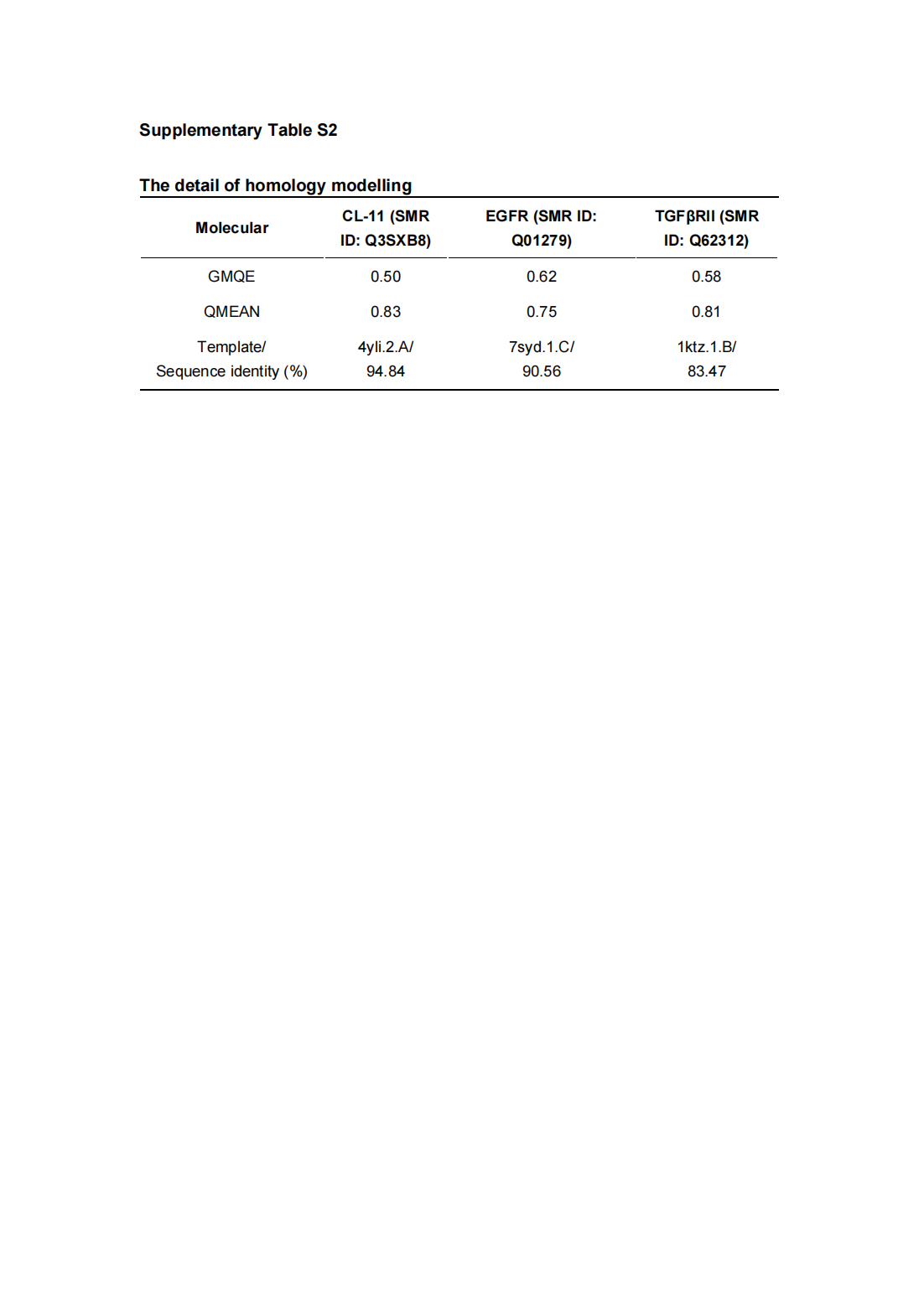

Supplement: Supplementary file 6 [file Image6.tif]
